# Association of the Gene Polymorphisms IFN-γ +874, IL-13 −1055 and IL-4 −590 with Patterns of Reinfection with *Schistosoma mansoni*

**DOI:** 10.1371/journal.pntd.0000375

**Published:** 2009-02-10

**Authors:** Michael R. Gatlin, Carla L. Black, Pauline N. Mwinzi, W. Evan Secor, Diana M. Karanja, Daniel G. Colley

**Affiliations:** 1 University of Georgia, Athens, Georgia, United States of America; 2 Kenya Medical Research Institute, Kisumu, Kenya; 3 Centers for Disease Control and Prevention, Atlanta, Georgia, United States of America; Case Western Reserve University School of Medicine, United States of America

## Abstract

**Background:**

The immunologic findings that most consistently correlate with resistance in human schistosomiasis are high levels of IgE and low levels of IgG4. We have genotyped gene and promoter polymorphisms of cytokines associated with regulation of these isotypes in a cohort of men occupationally exposed to *Schistosoma mansoni* in western Kenya and evaluated their patterns with respect to resistance and susceptibility to reinfection after treatment and cure with praziquantel (PZQ).

**Methodology/Principal Findings:**

In this cohort, polymorphisms in IL-4 (−590T high IgE), IL-13 (−1055T high producer) and IFN-γ (+874A high producer) demonstrated several correlations with resistance to reinfection. Resistance to reinfection was significantly correlated with the heterozygous IL-4 −590 genotype C/T (OR 3.5, [CI 1.2, 10.2]) compared to T/T. Among men with a homozygous IL-13 genotype CC/TT, having a T allele at the IFN-γ +874 position increased the odds of resistance relative to individuals with the IFN-γ +874 A/A genotype (OR = 17.5 [CI 3.0, 101.5]). Among men with homozygous A/A IFN-γ genotype, the heterozygous IL-13 genotype C/T was associated with resistance relative to the homozygous C/C or T/T genotypes (OR = 22.5 [CI 3.5, 144.4]). No increases in odds of resistance were found in relation to the IL-13 genotype among those with a T allele in the IFN-γ gene or in relation to the IFN-γ genotype among those with a heterozygous IL-13 genotype. Calculation of the attributable proportion of resistance showed a significant synergistic interaction between IL-13 −1055 C/T and IL-4 −590 C/T.

**Conclusions:**

The identified polymorphisms do not by themselves confer resistance or susceptibility, but we propose that these genotypes allow the resistant phenotype to be developed and expressed upon suitable immune exposure. Based on the literature, these polymorphisms contribute to the regulation of their respective cytokines, likely leading to downstream differences in the production and interrelationships of critical defense mechanisms.

## Author Summary

Approximately 200 million people have schistosomiasis in parts of Africa, South America, the Middle East, the Caribbean and Asia. Several studies of multiple treatments and reinfections indicate that some people develop resistance to reinfection. Of all the immunologic findings associated with such studies, the most consistent observation is that resistance (usually defined as lower levels of infection upon reinfection) correlates with high IgE and low IgG4 antibodies against schistosome antigens. Our studies test whether single nucleotide polymorphisms residing in the gene or promoter regions of cytokines pivotal in controlling production of these antibody isotypes are different amongst those that develop resistance to reinfection as opposed to those that do not. Through genotyping of these polymorphisms in a cohort of occupationally exposed car washers, we found that men with certain genotypic patterns of polymorphisms in IL-4, IFN-γ, and IL-13 were significantly more likely to be resistant to reinfection than those with different patterns. These data provide initial insights into the potential genetic foundation of propensities of people to develop resistance to reinfection by schistosomes, and offer a basis for further molecular studies of how these polymorphisms might work at the transcriptional and gene product level in cells stimulated by schistosome antigens.

## Introduction

There have been many studies of resistance to schistosome infections in humans following treatment and reinfection. Such studies involve documentation of cases of schistosomiasis, their treatment and cure, and examinations at a later date to see if reinfections occurred [1],[2],[3],[4]. Of all the immunologic findings associated with these investigations, the most consistent observation is that resistance (usually defined as lower levels of infection) correlates with high IgE and low IgG4 antibodies against schistosome antigens [5],[6],[7],[8],[9],[10],[11],[12],[13],[14],[15],[16],[17],[18]. Other studies have reported that production of IFN-γ and IL-5 to schistosome antigens are also correlated with resistance [19],[20],[21],[22].

A genetic region (SM1) has been identified that shows a strong positive correlation to the level of infection in humans [23]. SM1 maps to chromosome 5 in the 5q31–q33 region that contains several genes associated with immune responses [24],[25]. These genes code for proteins that are associated with the regulation of Th2-type responses such as IL-3, IL-4, IL-5, IL-9, and IL-13 and IgE. Polymorphisms in these cytokines that lead to an increase or decrease in cytokine levels could influence the antibody isotypes and cellular interactions that in turn may contribute to resistance or susceptibility of individuals to reinfection with schistosomiasis.

There have been contradictory reports on the effect the IL-4 −590 C/T (rs 2243250) polymorphism has on IgE levels in different settings. One group found that infants with a IL-4 −590 C allele had a higher risk of elevated IgE in their cord blood [26]. However, it was also reported that total IgE levels were significantly elevated in children with severe malaria carrying the −590T allele [27]. It is well known that IL-4 plays an important role in IgE class switching [28] and reports that the −590 C/T polymorphism are associated with differing amounts of IgE made it a candidate for this study.

Kouriba, et al. reported that IL-13 −1055C (rs 1800925) and −591A (rs 2069743) were associated with the upper 10% infection levels in individuals infected with *S. haematobium* (susceptibility) [29]. Van der Pouw Kraan et al. described an NF-AT binding site at IL-13 -1055 that showed increased binding of nuclear proteins with the T allele [30]. Furthermore, it was reported that transcription of the IL-13 -1055T allele was enhanced in Th2 polarized CD4+ cells, but not in nonpolarized (i.e., Th0) CD4+ T cells [31]. Thus, the IL-13-1055 polymorphism, and possibly −591, could be important in resistance to *S. mansoni*. The R130Q SNP (G/A) (rs 20541) in IL-13 causes a replacement of arginine with glutamine in α helix D, a region involved in IL-13 interactions with IL-13 receptors [32]. The glutamine variant has been associated with increased levels of total serum IgE [33] and atopy [34]. It has also been shown that soluble IL-13Rα2 (an IL-13 decoy receptor) neutralizes the arginine variant more effectively than the glutamine variant [32]. This may suggest a possible feedback mechanism that could account for increased total serum IgE in the glutamine variant.

While most studies have found that Th2 responses are associated with resistance to reinfection with schistosomiasis, other studies found that IFN-γ production correlates to resistance [19],[20],[21]. Pravica et al. showed that the +874T (rs 2430561) allele corresponds with high production of IFN-γ and that the A/T site coincides with a putative NF-kappa B binding site [35]. These studies prompted us to include the IFN-γ +874 polymorphism in our study.

IL-10 has been shown to influence IgE and IgG4 production [36]. Differential production of IL-10 has been shown to be associated with different allelic variants of the IL-10 promoter (−1082 A/G [rs 1800896], −819 T/C [rs 1800871], and −592 A/C [rs 1800872]). The different haplotypes that have been associated with production of IL-10 are as follows: “high” IL-10 producer haplotype (GCC/GCC), “intermediate” producer haplotypes (GCC/ACC, GCC/ATA), and “low” producer haplotypes (ATA/ATA, ACC/ATA, ACC/ACC) [37]. Indeed, the ATA/ATA haplotype has been associated with increased eosinophil counts and circulating IgE in adult asthma when compared to the other possible haplotypes [38]. However, the group that first described these polymorphisms conclude that high IL-10 production was dependant on having a G in the −1082 position independent of the −819 and −592 polymorphisms [39]. Furthermore, Ruess et al. has shown that −1082A binds PU.1, which can inhibit gene transcription.

Based on these and other studies, we examined possible relationships between single nucleotide polymorphisms in the IL-4 (−590 C/T), IL-13[(−1055 C/T), (−591A/G), (R130Q G/A)], IL-10 (−1082 A/G; −819 C/T; −592 C/A), and IFN-γ (+874A/T) genes or promoters in relation to resistance and susceptibility to schistosomiasis of a cohort of occupationally exposed adult men many of whom we have studied longitudinally for as long as 12 years [40],[41].

## Materials and Methods

### Study participants

The field site for this study was the western Kenyan city of Kisumu on the shores of Lake Victoria. *S. mansoni*-infected *Biomphalaria sudanica* snails have been identified (data unpublished) in the area around the exposure site. The study participants were all car washers occupationally exposed to *S. mansoni* as they stood in Lake Victoria, using its waters to wash cars driven into the shallow areas of the lake. This study was performed on a total of 87 car washers (all adult men). However, it was not possible to use all data from every individual in every analysis; some epidemiologic data regarding infections, cures or reinfections were insufficient or incomplete.

This investigation was approved by the Institutional Review Boards of the University of Georgia and the Centers for Diseases Control and Prevention, the Scientific Steering Committee of the Kenya Medical Research Institute (KEMRI), and the National Ethics Review Committee of Kenya. After obtaining written informed consent and enrolling the participants, we examined their stools for *S. mansoni* eggs and for other helminth ova by the modified Kato-Katz method (Vestergaard-Frandsen, Denmark). In most instances, this involved 2 slides per stool specimen from 3 stool specimens over a one week period. The participants who were positive for *S. mansoni* were treated with 40 mg/kg praziquantel (PZQ); men positive for other soil-transmitted helminth eggs were treated with 400 mg albendazole. Individuals' stools were checked 6 weeks after treatment and the men were retreated if still egg positive. Upon becoming egg negative, they were then followed by stool examination every 4 weeks to determine their time to reinfection as a means of determining their relative resistance to reinfection (see below).

### DNA preparation

White blood cell-containing buffy coats were separated using the Ficoll-hypaque technique from blood obtained for immunological assays. The buffy coats were stored at −20°C and DNA was isolated at KEMRI/CGHR laboratories using the Wizard® Genomic DNA Purification Kit from Promega. DNA from 300 µl of buffy coat for each car washer was isolated per the manufacturer's instructions. Dried DNA pellets were then transported to Athens, Georgia, USA for genotyping.

### PCR reactions

PCR reactions of IL-13 and IL-4 SNPs were performed on a PTC-200 DNA Engine from MJ Research. PCR of the IL-13 −1055 C/T was conducted in a 50 µl reaction containing 100 ng DNA, 5 µl of 1× Qiagen PCR buffer, 0.5 µM of each dNTP, 0.4 µM of each primer, 1 mM MgCl_2_, and 2.5 U of *Taq* polymerase (Qiagen). The following sequences were used: forward primer, 5′-ATGCCTTGTGAGGAGGGTCAC; reverse primer, 5′-CCAGTCTCTGCAGGATCAACC [42]. Initial denaturation was performed at 95°C for 3 min followed by 30 cycles of PCR with the following conditions: 95°C for 30 sec, 62°C for 30 sec for annealing, 72°C for 1 min, and a final 72°C for 3 min. PCR of the IL-13 −591 A/G was conducted in a 50 µl reaction containing 100 ng DNA, 5 µl of 1× Qiagen PCR buffer, 0.5 µM of each dNTP, 0.4 µM of each primer, 3 mM MgCl_2_, and 2.5 U of *Taq* polymerase (Qiagen). Initial denaturation was performed at 94°C for 5 min followed by 34 cycles of PCR with the following conditions: 94°C for 1 min, 61°C for 45 sec for annealing, 72°C for 45 sec, and a final 72°C for 3 min. The following sequences were used: forward primer, 5′-CCAGCCTGGCCCAGTTAAGAGTTT; reverse primer, 5′-CTAATTCCTCCTTGGCCCCACT [29]. PCR of the IL-13 +130 G/A was conducted in a 50 µl reaction containing 100 ng DNA, 5 µl of 1× Qiagen PCR buffer, 0.5 µM of each dNTP, 0.4 µM of each primer, 1 mM MgCl_2_, and 2.5 U of *Taq* polymerase (Qiagen). Initial denaturation was performed at 94°C for 5 min followed by 34 cycles of PCR with the following conditions: 94°C for 1 min, 60°C for 45 sec for annealing, 72°C for 45 sec, and a final 72°C for 3 min. The following sequences were used: forward primer, 5′-TGGCGTTCTACTCACGTGCT; reverse primer, 5′-CAGCACAGGCTGAGGTCTAA [43]. PCR of the IL-4 −590 C/T was conducted in a 50 µl reaction containing 100 ng DNA, 5 µl of 1× Qiagen PCR buffer, 0.5 µM of each dNTP, 0.4 µM of each primer, 1 mM MgCl_2_, and 2.5 U of *Taq* polymerase (Qiagen). Initial denaturation was performed at 95°C for 5 min followed by 31 cycles of PCR with the following conditions: 94°C for 30 sec, 59°C for 30 sec for annealing, 72°C for 30 sec, and a final 72°C for 3 min. The following sequences were used: forward primer, 5′-ACTAGGCCTCACCTGATACG; reverse primer, 5′-GTTGTAATGCAGTCCTCCTG [44].

### Genotyping

Purified PCR products of the IL-13 −1055 C/T and IL-4 −590 C/T PCR reactions were sequenced using the reverse primers for each and the forward primers for IL-13 +130 G/A and IL-13 −591 A/G on an ABI 3100 by the Office of Research Services at The University of Georgia. The polymorphisms IL-10 −1082, IL-10 −819, IL-10 −592, and IFN-γ +874A/T were genotyped using sequence-specific primers (SSP) in The Cytokine Genotyping Tray (One Lambda; Canoga Park, CA) as per the manufacturer's instructions.

### Resistance

Resistance is based on the number of cars washed from the time of a successful cure until the next reinfection. For all participants, the number of cars washed between each cure and reinfection over the entire duration of the study was plotted and the patterns examined. Two dominant patterns emerged, with participants either becoming reinfected after washing approximately the same number of cars between each cure and reinfection or participants washing progressively more cars between successive cure-reinfection intervals. The majority of those in the former group (classified as “susceptible”) became reinfected after washing approximately 250–300 cars regardless of how many times they were cured and reinfected. Those men who demonstrated a pattern of increasing numbers of cars washed before subsequent reinfections were classified as “developing resistance” during this study. All men classified as “developing resistance” eventually washed at least 450 cars before reinfection after being followed for at least 3 cure-to-reinfection intervals. Some men (classified as “initially resistant”) washed at least 450 cars after the initial cure and continued to wash a high number of cars before each reinfection. For analysis purposes, men classified as “developing resistance” and “initially resistant” were grouped into a single “resistant” category because frequencies of genotypes did not differ significantly between these two groups, and conceptually these groups indicate either the existence of established resistance or the ability to develop resistance. Resistance data are based on a mean follow-up time of 7.5 years (range 0.9–12.2 yrs) months and a mean of 7 cure-to-reinfection intervals. Men were excluded from the analysis when the number of cars washed between each cure and reinfection could not be classified into a particular pattern (3 men) or they had insufficient follow up data (16 men) for accurate classification.

### Statistical methods

Odds ratios (ORs) and corresponding 95% confidence intervals (CIs) for the association between resistance and each genotype were calculated using univariate analyses and in a multivariate logistic regression model containing variables for all three genotypes. To test for interactions between genotypes, categorical interaction terms with 4 levels were created for each possible two-way interaction between the 3 dichotomized genotypes. The combination of alleles with the lowest frequency of resistant subjects was considered the referent category in each set of terms. Separate logistic regression models were run for each series of two-way interaction terms. Inclusion of a term for the third genotype did not appreciably change the results of any of the interaction models and was thus ultimately not included in any of the reported analyses of interactions.

Interactions were assessed on both a multiplicative and additive scale. Multiplicative interactions are indicative of the need to estimate stratum-specific effects for the combination of two genotypes, rather than a single estimate for each genotype, in order to improve the fit of the model to the data [45]. Multiplicative interactions were assessed by testing the significance of an interaction term between two genotypes in the logistic regression models by the Wald test. As many researchers believe that departure from additivity is a better indicator of biologic interaction than departure from multiplicativity [45],[46], we also calculated the attributable proportion (AP) of resistance due to interactions between two genotypes. A positive AP is indicative of synergy between the two genotypes, while a negative AP indicates antagonism [47]. APs and corresponding 95% CIs for each of the two-way interactions between genotypes were calculated based on the output from the logistic regression models using the code provided by Andersson et al [48] based on the methodology described by Hosmer and Lemeshow [49]. All analyses were performed with SAS version 9.1.

## Results

### Distribution of genotypes and resistance

The distribution of genotypes for each polymorphism and frequency of resistance in each genotype are given in Table 1. A higher percentage of car washers with a T allele (T/T or T/A) at IFN-γ +874 are resistant (T/A 65.2% and T/T 77.8%) than men that are A/A homozygous (38.9%). For subsequent analyses, men with T/A and T/T at the IFN-γ +874 position were grouped as the occurrence of resistance for each was significantly higher when compared to homozygous individuals (A/A) and they did not differ from each other. Among men heterozygous (C/T) at the IL-13 −1055 position, 76.0% were resistant to reinfection; whereas only 41.9% of C/C and only 42.9% of T/T individuals were resistant to reinfection (Table 1). Both homozygous genotypes for IL-13 −1055 were grouped for the univariate analysis as the occurrences of resistance in either of those genotypes were substantially lower than in the heterozygous group and they did not differ from each other. The frequency of homozygous C/C at IL-4 −590 was not high enough in our cohort of men to allow analysis. However, men heterozygous (C/T) at IL-4 −590 represent a higher percentage of resistance (70.8%) than men that are T/T homozygous (40.9%). No overt differences are seen in the proportions of the different genotypes of IL-10 (−1082; −819; −592) promoter SNPs in relationship to resistance or susceptibility to reinfection, and as expected due to linkage disequilibrium, SNPs at IL-10 −819 and IL-10 −592 segregate together.

**Table 1:** IFN-γ +874, IL-13 −1055, IL-13 +130, IL-13 −591, IL-4 −590, IL-10 −1082, IL-10 −819 and IL-10 −592 genotype distributions and percentage of resistance.

Genotype	Total N	N (% susceptible)	N (% resistant)
**IFN-γ +874**			
A/A	36	22 (61.1%)	14 (38.9%)
T/A	23	8 (34.8%)	15 (65.2%)
T/T	9	2 (22.2%)	7 (77.8%)
**IL-13 −1055**			
C/C	31	18 (58.1%)	13 (41.9%)
C/T	25	6 (24%)	19 (76%)
T/T	7	4 (57.1%)	3 (42.9%)
**IL-13 +130**			
A/A	7	2 (28.6%)	5 (71.4%)
A/G	23	8 (34.8%)	15 (65.2%)
G/G	33	18 (54.5%)	15 (45.5%)
**IL-13 −591**			
A/A	55	26 (47.3%)	29 (52.7%)
A/G	8	2 (25%)	6 (75.0%)
**IL-4 −590**			
C/C	1	0	1 (100%)
C/T	24	7 (29.2%)	17 (70.8%)
T/T	44	26 (59.1%)	18 (40.9%)
**IL-10 −1082**			
A/A	38	21 (52.6%)	18 (47.4%)
A/G	27	12 (44.4%)	15 (55.6%)
G/G	4	1 (25%)	3 (75.0%)
**IL-10 −819**			
C/C	14	5 (35.7%)	9 (64.3%)
C/T	39	17 (43.6%)	22 (56.4%)
T/T	16	11 (68.7%)	5 (31.3%)
**IL-10 −592**			
C/C	14	5 (35.7%)	9 (64.3%)
C/A	39	17 (43.6%)	22 (56.4%)
A/A	16	11 (68.7%)	5 (31.3%)

### Polymorphisms in IL-13, IFN-γ, IL-4, and IL-10 and resistance

Resistance was significantly associated with the T allele at IFN-γ +874 (TT and TA) when compared to the homozygous A/A individuals (OR 3.5 [CI 1.3, 9.4]) (Table 2). Car washers heterozygous (C/T) at the IL-13 −1055 position also showed a significant correlation with resistance to reinfection when compared to homozygous (C/C and T/T) car washers (OR 4.4 [CI 1.4, 13.4]) (Table 2), and heterozygousity (C/T) at the IL-4 −590 position correlated significantly with resistance when compared to homozygous (T/T) car washers (OR 3.5 [CI 1.2, 10.2]) (Table 2). These associations remained significant when all three genotypes were included in a multivariate analysis, indicating an independent association between resistance and each of the 3 genotypes (Table 2). For IL-13 −591, there was no significant difference in resistance between the A/A and A/G genotypes (Table 1, p = 0.2399). Having an A allele at the IL-13 +130 position was associated with a modest increase in resistance relative to the homozygous G/G genotype (OR = 2.4 [0.9, 6.7]). However, when IL-13 +130 and IL-13 −1055 were evaluated simultaneously in a logistic regression model, the association between resistance and IL-13 +130 disappeared (OR = 1.4 [0.4, 4.5]), while IL-13 −1055 remained significantly associated with resistance. This suggests that the observed relationship between resistance and IL-13 +130 was spurious and likely due to the close association between IL-13 +130 and IL-13 −1055. Therefore, we only included the IL-13 −1055 polymorphism in further analyses.

**Table 2:** Odds ratios for the associations between individual genotypes and resistance to reinfection by univariate and multivariate analysis.

Genotype	Univariate		Multivariate*	
	OR (95% CI)	p-value	OR (95% CI)	p-value
IL-13 −1055 (C/T vs CC/TT)	4.4 (1.4, 13.4)	0.0086	6.3 (1.5, 26.0)	0.0109
IFN-γ +874 (TT/TA vs A/A)	3.5 (1.2, 9.4)	0.0145	9.0 (2.0, 38.3)	0.0030
IL-4 −590 (C/T vs T/T)	3.5 (1.2, 10.2)	0.0192	4.3 (1.1, 17.4)	0.0419

*: adjusted for the other 2 genotypes.

We found a relationship of borderline significance with IL-10 −819 or −592 (any C versus homozygous G/G or A/A): (OR = 3.1 [0.9, 10.2], p = 0.0577) and resistance to reinfection. These two (−819 and −592) polymorphisms are in tight linkage disequilibrium; hence the results are interchangeable between the two. The OR remained virtually unchanged in a model controlling for the other three significant genotypes, indicating that this possible low-grade association between resistance and the IL-10 polymorphism was not confounded by its association with another genotype. There were no significant additive or multiplicative interactions between IL-10 −819 or −592 and any of the IL-4, IL-13 or IFN-γ polymorphisms. No significant associations were found with IL-10 −1082 with resistance or susceptibility (data not shown).

### Associations between genotype and resistance within categories of genotype interactions

Combination of the IL-13 −1055 and IFN-γ +874 genotypes that were independently associated with resistance did not increase the odds of being resistant over having either one of these genotypes alone. However, although all combinations of IL-13 and IFN-γ genotypes had comparable odds of resistance when compared to the reference group (Table 3), effect modification was seen between the IL-13 and IFN-γ genotypes. Among men with a homozygous IL-13 genotype, having a T allele at the IFN-γ +874 position increased the odds of resistance relative to those with the A/A genotype (OR = 17.5 [CI 3.0, 101.5]) (Table 3). There was no association between IFN-γ genotype and resistance among men with the heterozygous C/T IL-13 genotype. Likewise, among men with homozygous A/A IFN-γ genotype, the heterozygous IL-13 genotype was associated with resistance relative to the homozygous C/C or T/T genotypes (OR = 22.5 [CI 3.5, 144.4]) (Table 3), while no association between IL-13 and resistance was seen among men with a T allele in the IFN-γ gene.

**Table 3:** Odds ratios for the associations between genotype and resistance within categories of genotype interaction.

Interaction categories	Odds Ratio (95% CI)	Measures of interaction	n (%) resistant
**IL-13 and IFN-γ**			
IL-13 CT, IFN-γ TA/TT	26.3 (3.0, 226.6)	AP+ = −0.49 (−3.09, 2.12)	7 (77.8)
IL-13 CT, IFN-γ AA	22.5 (3.5, 144.4)	Wald p-value* = 0.047	12 (75.0)
IL-13 CC/TT IFN-γ TA/TT	17.5 (3.0, 101.5)		14 (70.0)
IL-13 CC/TT, IFN-γ AA	Ref		2 (11.8)
**IL-13 and IL-4**			
IL-13 CT, IL-4 CT	20.1 (2.3, 176.0)	AP+ = 0.90 (0.64, 1.15)	13 (92.9)
IL-13 CT, IL-4 TT	1.9 (0.5, 7.6)	Wald p-value* = 0.105	6 (54.5)
IL-13 CC, IL-4 CT	1.3 (0.3, 5.6)		4 (44.4)
IL-13 CC/TT, IL-4 TT	Ref		11 (39.3)
**IL-4 and IFN-γ**			
IL-4 CT, IFN-γ TA/TT	14.9 (2.2, 100.7)	AP+ = 0 (−1.75, 1.75)	7 (77.8)
IL-4 TT, IFN-γ TA/TT	7.4 (1.9, 30.0)	Wald p-value* = 0.240	14 (63.6)
IL-4 CT, IFN-γ AA	8.5 (1.8, 39.2)		10 (66.7)
IL-4 TT, IFN-γ AA	Ref		4 (19.1)

**+:** AP = attributable proportion.

*: Wald test for interaction term in logistic regression model.

Men with a combination of the heterozygous C/T alleles for IL-13 −1055 and IL-4 −590 genes had more than a 20-fold increased odds of resistance relative to those with homozygous alleles at both genes (OR = 20.1 [CI 2.3, 176.0]) (Table 3). Men who were heterozygous for only one of the IL-13 or IL-4 genes were not more likely to be resistant compared to men with both homozygous alleles (Table 3). A significant additive interaction between IL-13 and IL-4 genotypes was detected, with 90% of the resistance among those with heterozygous alleles at both genes attributable to the interaction between the two genes (AP = 0.90 [0.64, 1.15]). In contrast, no significant additive or multiplicative interactions were detected between IL-4 −590 and IFN-γ +874 (Table 3).

## Discussion

The car washers in this study are part of a longitudinal study that dates back 12 years [40]. Their exposure (water contact) to infection with *S. mansoni* is documented by the number of cars washed for payment purposes, providing us with a unique, reliable means to quantify an individual's exposure in an active transmission site over time. This longitudinal field setting allows us to account for water contact as a variable and pose some interesting immunologic and genetic questions with regard to what influences may play a role in determining resistance to reinfection after treatment.

We found significant correlates between resistance to reinfection with *S. mansoni* and the heterozygous (C/T) IL-13 −1055 genotype, any T allele in the IFN-γ +874 genotype, and the heterozygous (C/T) in the IL-4 −590 genotype by univariate analysis. Furthermore, these associations remained significant when all three genotypes were included in a multivariate analysis, indicating independent associations between resistance and each of the 3 genotypes. However, our data suggest that having a combination of the IL-13 C/T and an IFN-γ T allele at +874 does not provide increased odds of being resistant over having just one of these genotypes. Instead, both IL-13 C/T and any IFN-γ T allele demonstrated very high odds of being resistant when compared to the reference group. Our data did, however, show a significant synergistic effect between the IL-13 −1055 C/T and IL-4 −590 C/T genotypes. Thus, the proportion of resistant men seen with a combination of these two cytokine genotypes was much greater than that seen with the sum of the separate effects of IL-13 −1055 C/T and IL-4 −590 C/T on resistance. We interpret these data to indicate that individuals heterozygous at the IL-13 −1055 and IL-4 −590 position are more likely to require fewer reinfections and treatments to become resistant to reinfection than individuals who are homozygous at either position.

Due to our small sample size, our interaction analysis resulted in wide confidence intervals and we were unable to evaluate 3-way interactions between groups. Also, because car washing is an exclusively male profession at this study site, the associations in this study apply to men and cannot necessarily be generalized to women. However, these analyses provide interesting findings in a situation that provides exceptional exposure data that are not generally available in studies of resistance to reinfection. Clearly our findings need to be investigated further in larger cohorts. However, the current data provide initial insights into the potential genetic foundation of propensities of people to develop resistance to reinfection by schistosomes, and they offer a basis for further molecular studies of how these polymorphisms might work at the transcriptional and gene product level.
